# Spatio-temporal distribution and classification of utilization of urban bare lots in low-slope hilly regions

**DOI:** 10.1371/journal.pone.0246746

**Published:** 2021-02-19

**Authors:** Qi Cao, Manjiang Shi

**Affiliations:** Department of Civil Engineering and Architecture, Southwest University of Science and Technology, Mianyang, Sichuan Province, China; Northeastern University (Shenyang China), CHINA

## Abstract

Urban bare lots are persistent phenomena in urban landscapes in the course of urbanization. In the present study, we examined the spatio-temporal distribution of urban bare lots in low-slope hilly areas, and to assess the major pathways by which they are generated and later re-transformed for exploitation. We extracted land use and land cover (LULC) change information and analyzed spatio-temporal distribution characteristics of urban bare lots using Landsat TM/OLI series remote sensing images. Subsequently, we proposed an index system for their evaluation and classification, and identified five types of urban bare lots. Urban bare lot quantity and distribution are closely correlated with human activity intensity. Stakeholders should consider the multiple effects of location, topography, landscape index, transportation, service facilities, and urban planning in urban bare lot classification activities for renovation and re-transformation.

## Introduction

Urban bare lots refer to properties with surfaces that comprise mainly soil and minimal vegetation cover [1, 2], which is a pressing issue in many cities worldwide [3]. In cities with a population of about 1 million, urban vacant land accounts for approximately 19% to 25% of the urban area [4]. Concern over urban bare lots stems from the phenomenon of "counter-urbanization", but it was not until the 21st century that the issue gradually became an important part of urban land use research. A variety of urban processes, including decentralization due to demographic changes, urban sprawl, de-industrialization, and people’s preferences for new types of residential choices, have led to increased vacancy rates in urban areas [5].

Human activities directly or indirectly lead to the degradation of surface vegetation and the formation of bare surfaces through the expansion of residential areas, open-pit mining, urban infrastructure construction, etc. The alarming growth rate of urban bare lots, whether in shrinking or sprawling cities in many countries [6, 7], aggravates urban dust [8] and air particle pollution [9, 10], as well as the deterioration of the urban ecological environment [11], even to the extent of reducing the quality of human life [12]. Urban bare lots also symbolize urban disinvestment and decay, and consequently are very vulnerable to being used for marginal activities [13]. Nevertheless, urban bare lots can provide more economic, ecosystem, and cultural services than other impervious surfaces in urban areas, if well utilized [14]. It is important to reduce the sizes and increase the utilization ratio of urban bare lots to alleviate the conflict between supply and demand for urban land and improve the urban ecological environments [15].

In Europe and North America, regeneration of brownfield land is an important government policy [16]. Regeneration into green spaces, in particular, has been used as a way of reversing social and environmental decline, with typical benefits including increased flood retention capacity, temperature regulation, more habitats for wildlife, enhancement of interactions within local communities, and providing more spaces for play and recreation. O’Brien [17] has demonstrated that trees, forests, and green spaces enhance the quality of life of individuals, in addition to their physical, psychological, and social wellbeing. Green spaces, for example, facilitate interactions among residents within communities and can improve social relations and foster a sense of belonging [18]. Green spaces also have an aesthetic value, which is an important ecological service to society. Social benefits associated with the regeneration of brownfield lands into green infrastructure also include relaxation and ‘quiet recreation’, in addition to greater opportunities to walk, cycle [19], and experience nature [20]. Setting up new green spaces on brownfield land can provide urban residents access to natural green spaces close to their residences [21].

Regenerating a brownfield site into a green space brings the land back into use and community forest land could serve multiple functions, such as play areas and community gardens. Such activities can also enhance the provision of ecosystem services such as temperature regulation (e.g. urban cooling) and increase the overall vegetation cover. Most sites aspire to serve multiple benefits and efforts in the short term to create green spaces on brownfield lands can lead to long term benefits as the space develops and more people begin to exploit its benefits [22].

The Chinese government has replaced multiple related plans (such as urban master plan and detailed urban construction plan) with territorial spatial planning efforts, to facilitate the effective resolution of conflicts among various land-use planning activities [23]. However, such policies are yet to comprehensively address the conflicts among the different land-use functions [24]. Therefore, analyzing the spatio-temporal distribution characteristics of urban bare lots and proposing appropriate approaches of renovating and re-utilizing urban bare lots based on carrying capacity analyses and land-use suitability evaluations could optimize urban land-use patterns and conserve cultivated and forest lands through effective coordination among pre-existing land-use plans, land-use status quo, and urban growth processes.

Hilly areas account for approximately two-thirds of China’s land area and play an important role in the regulation of regional climate and environment, the maintenance of biodiversity, water conservation, and other aspects of ecological services [25]. However, due to disorderly expansion leads to the formation numerous exposed surfaces in hilly urban areas in the course of urbanization. Because of their scattered distribution and small areas, urban bare lots are often ignored during the monitoring, research, and LULC planning in urban locations [26, 27]. In addition, in the current land use classification system in China, urban bare lots are classified under unused land, while traditional research on urban bare lots has mainly focused on the physical characteristics and functions of urban bare lots such as soil-water-heat exchange [28, 29], with hardly any research exploring the formation, transformation, redevelopment, and utilization of urban bare lots.

With the emergence of the Smart City concept [30–33], vacant and abandoned land in cities have attracted the attention of researchers and stakeholders globally [34–38]. To explore and promote the ecosystem services of urban vacant land, scholars in western nations have conducted research on urban greening, urban agriculture, and expansion/reconstruction of public spaces [39–42]. Research on urban bare lots in China could enhance our understanding of the transformation and utilization dynamics of vacant land in developed countries.

Land mobility seems to be an indispensable component of the man-land relationship [43], especially in the context of human migration that facilitates redistribution and re-utilization, and urban bare land is no exception. A variety of urban processes, including urban sprawl and de-industrialization, have led to increased vacancy rates in urban areas, with numerous properties being degraded into urban bare lots. Countries globally are committed to the evaluation, identification, regeneration, and reuse of urban bare, idle, and brownfield lands. However, few studies have explored the classification of urban bare land, and the existing classification methods of bare land have not elaborated the classification criteria [23, 24]. Therefore, the research question is: what is the objective basis of urban bare land classification transformation and reuse? This study attempts to determine a relatively unified and cross-referenced quantitative evaluation basis for urban bare land under different natural and cultural characteristics. In other words, quantitative analysis guides the standard of "what kind of urban bare land should be transformed into what kind of urban functional land".

Hardly any studies have integrated the spatio-temporal distribution of urban bare lots in their classification and evaluation activities. Therefore, the objective of the present study was to assess the dominant pathways by which urban bare lots are generated and later transformed to provide beneficial services based on their spatio-temporal distribution and regeneration in low-slope hilly areas. The specific objectives include: (1) analysis of the spatial and temporal distribution and land transfer characteristics of urban bare lots in the course of urbanization, (2) discussion of the classification and zoning methods of reconstruction and utilization of urban bare lots in order to identify adjustment in measures appropriate to the local conditions and improve the utilization rate of urban bare lots. Analyzing the spatio-temporal distribution of urban bare lots could facilitate the formulation of appropriate management strategies for the improvement or prevention of environmental deterioration and loss of natural resources. The results of the present are expected to provide reference for the transformation and effective exploitation of urban bare lots in the course of urbanization.

The goal of the present study is to present an indexing scheme that planning agencies can employ to scan large areas and determine which urban bare sites should be considered for further assessment and redevelopment. The index incorporates indicators for six dimensions, and it aggregates location specific variables into a mapping index that is visualized in a Geographic Information System (GIS) tool. We apply the index for the City of Mianyang and the State of Sichuan, China. The rest of this paper is organized as follows. The next section gives an overview of the related literature, which is followed by the data and methods section. The results section presents the spatial and temporal distribution and land transfer characteristics of urban bare lots based on the “hierarchical classification method” [1] and an analysis of reconstruction and utilization by evaluating of multiple factors influencing urban bare lots [44]. Finally, the discussion and conclusion section summarize our results and their implications for urban resilience planning.

## Data and methods

The main data sources are shown in Table 1. Remote sensing data from Landsat TM and OLI were downloaded from USGS website (https://earthexplorer.usgs.gov, Path = 130, Row = 38). The geospatial data used also included the Mianyang digital elevation model (DEM with spatial resolution of 30 m; https://search.asf.alaska.edu/), the road network and points of interest (POI). The latter contains 16,877 pieces of information in four categories, including business residence, shopping service, catering service, and accommodation service.

**Table 1:** Landsat images and auxiliary data used in this study.

Type		Time	Spatial resolution	Wind cover	Purpose
Remote sensing data source	Landsat TM	1999/6/12	Multispectral band 30 m	9%	Land use change information and inversion of surface temperature
	Landsat TM	2005/4/14	Multispectral band 30 m	0%	
	Landsat TM	2011/6/2	Multispectral band 30 m	0%	
	Landsat OLI/TIRS	2017/5/1	Multispectral band 30 m	4%	
			panchromatic band 15 m		
Auxiliary geospatial data	Mianyang digital elevation model (DEM)		30 m	0%	Terrain analysis
	POI data from website	2017/3	/	/	Density analysis
	Mianyang City central urban area layout planning (2010–2020)		/	/	Land spatial layout
Online data	Historical HD image from Google Earth	2017/10/1	0.5 m	0%	Verification of remote sensing interpretation accuracy
		2010/11/15			

### Remote sensing interpretation of LULC

**Fig 1:**
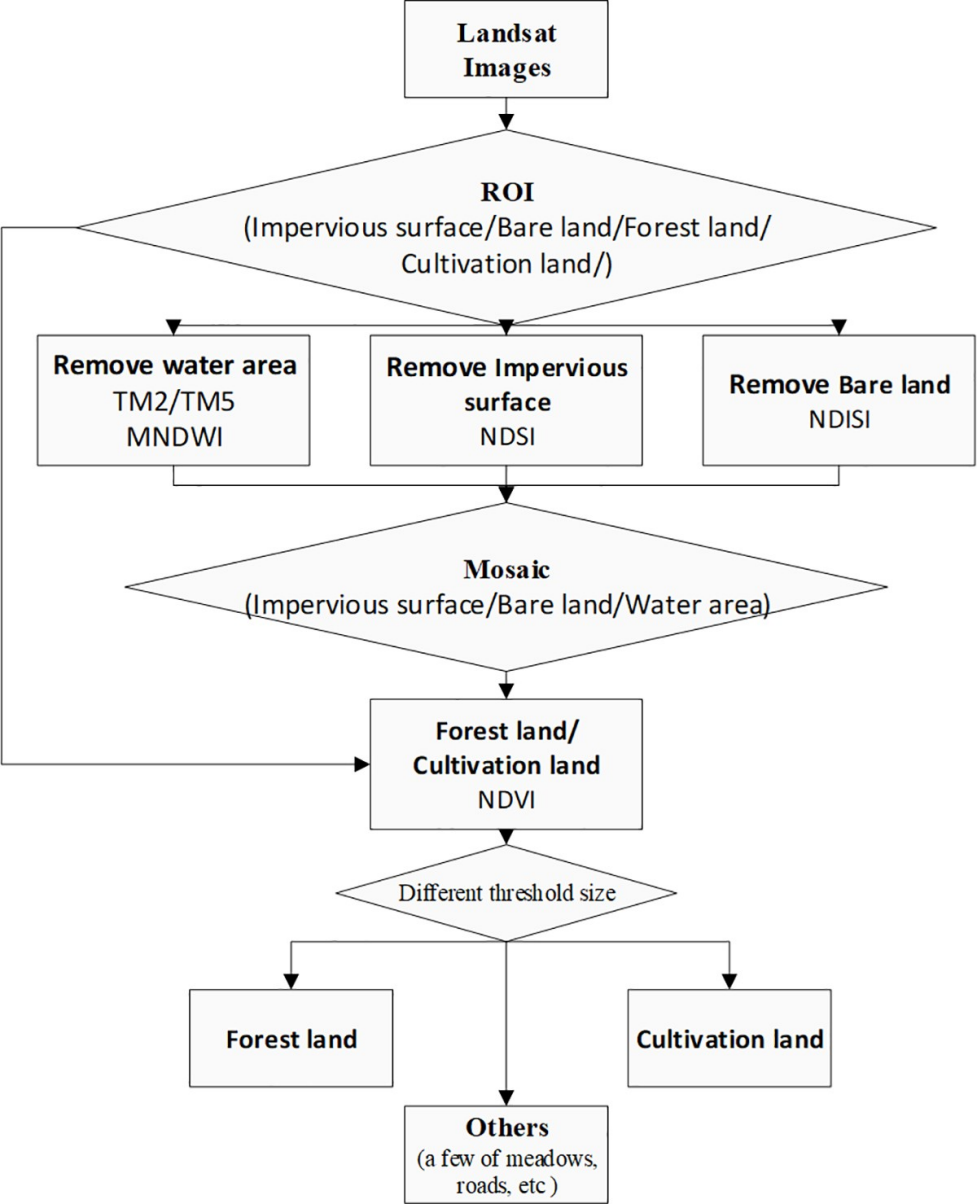
Hierarchical classification.

LULC information was interpreted by hierarchical classification [1] of four batches of Landsat TM/OLI series remote sensing images [45] (1999, 2005, 2011, and 2017). The procedure was used to divide LULC types into six categories: urban bare lots, urban impervious surface, cultivated land, forest land, surface water, and others (Fig 1). First, the urban bare lots were extracted, and then the urban impervious surfaces were extracted after processing the urban bare lot mask. Similarly, after extracting the urban impervious surface, the two types of land (urban bare lots and urban impervious surface) extracted are masked before extracting the third type of land. The procedure was repeated until all land use types were extracted. Among them, urban impervious surface and urban bare lot extraction was based on Normalized Difference Impervious Surface Index (NDISI) [46–48] and Normalized Difference Soil Index (NDSI) [49, 50] indices. Vegetation and surface water are relatively easy to extract because of obvious differences in spectral characteristics. Forest land and cultivated land were discriminated from one another based on an NDVI (Normalized Difference Vegetation Index) [51] threshold.

**Fig 2:**
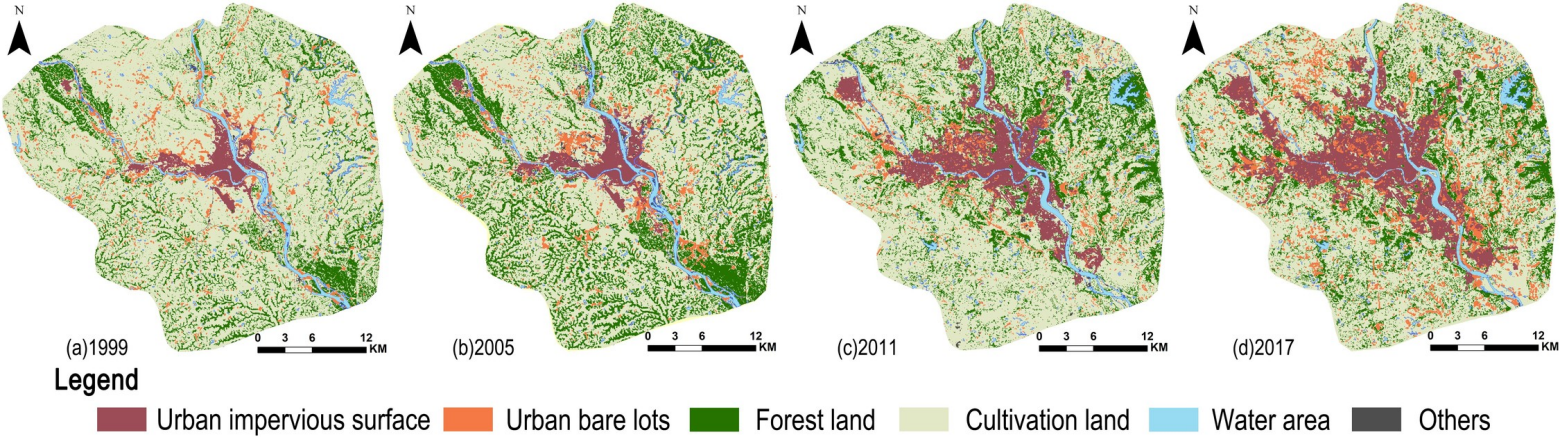
The LULC information base on Landsat TM/OLI images in the study area from 1999 to 2017.

The land use types assigned to the others category were obtained by erasing the area of the above five types of land from the whole study area using the erase tool in ArcMap 10.2 (ESRI, Redlands, California, USA), after verifying the accuracy of the lands assigned to the five types. Based on reference to historical HD images of the study area from Google Earth (Alphabet Inc., Mountain View, California, USA) in 2010–2017, interpretation accuracy verification selected the purest ROI (region of interest, ROI) as possible using Landsat TM and OLI data. The number of ROI for each land use type was not less than 100. Subsequently, the extracted information was compared with the ROI to ensure that the accuracy of each type of land was maintained at 90% or greater. The results are shown in Fig 2.

### Analysis of temporal and spatial changes in LULC and urban bare lots

#### Annual change rate and dynamic degree of urban bare lots

The equations to calculate the annual change rate (*Ki*) and dynamic degree (*Di*) [52] of *i*^*th*^ land are as follows:

$$K_{i}=\left(\frac{\Delta S_{i,t}}{S_{i}}\right)\times \frac{1}{t}\times 100\%$$ (1)

$$D_{i}=\left(\frac{\left|\Delta S_{i,t}\right|}{S_{a}}\right)\times \frac{1}{t}\times 100\%$$ (2)

where *S*_*i*_ is the total area of *i*^*th*^ land use type at the beginning of monitoring; *ΔS*_*i*,*t*_ is the net change area of the *i*^*th*^ land use type from the beginning to the end of monitoring; *t* is the time period; *S*_*a*_ is the total area of the study area.

#### Landscape pattern analysis of urban bare lots

The landscape pattern of urban bare lots from 1999 to 2017 was characterized by patch number, patch area, and patch shape [53] (Table 2). The equation for calculating mean shape index (MSI) is as follows:

$$MIS=0.25P/\sqrt{A}$$ (3)

Where *P* represents the total patch perimeter of urban bare lots and *A* represents the total patch area of urban bare lots.

**Table 2:** Landscape indexes for urban bare lots.

Landscape index	Distribution
Patch Number (*PN*)	Counted patches of urban bare lots in ArcMap.
Mean Patch Area (*MPA*)	The total patch area divided by the number of urban bare lots
Maximum Patch Area (*Max*.*PA*)	Counted maximum patch area of urban bare lots in ArcMap.
Standard Deviation of Patch Area (*MPASD*)	Calculated the standard deviation of patch area to measure the discrete degree of urban bare lots area.
Mean shape index (*MSI*)	With a square as the standard, if *MSI* = 1, the patch shape is close to being square or circular (*MSI* = 0.88), if *MSI* > 1, the patch shape is close to oval or rectangular.
Standard Deviation of Mean Shape Index (*MSISD*)	To measure the dispersion degree of *MSI* in urban bare lots, the larger the value is, the more irregular the shape is.

### Urban bare lot transformation and utilization classification

Urban bare lots can deliver fundamental ecosystem services with direct economic and social benefits [54], such as primary production (provisioning), climate regulation, carbon sequestration, and air-pollution removal (regulating). Urban bare lot greening improves neighborhood conditions, and initiatives that transfer vacant lots to neighborhood residents can return benefits to where they are most needed [55]. Before such initiatives can be undertaken, urban bare lots have to be identified and classified. Land use classification reflects the forms and functions of land use, and the modes and impacts of land use and transformation by human beings. It also facilitates the rationalization of the organization of land use and distribution of production according to local conditions [56].

Based on relevant literature across the globe, the transformation and utilization of urban bare lots can be divided into five categories. The first type of urban bare lots (such as those scattered in streets and communities) is distributed in and around urban areas and close to human settlements and is mainly used to build small parks and recreational grounds [57, 58], sidewalks, and other community public services and infrastructure [59, 60]. Although urban bare lot greening cannot substitute plant community restoration activities, it is a key step toward rebuilding plant communities and mitigating green space deficiencies in old neighborhoods [61, 62]. The second type of urban bare lots occupies relatively large areas and is associated with improved ecological backgrounds. Such bare lots can be used to build urban parks, urban ecological corridors, and to implement other types of urban green projects [63]. The third type of bare lots is located close to logistics, industrial parks, and construction sites, and can be used for the construction of urban infrastructure and public facilities (such as streets, squares, and parking lots) [11]. The fourth and fifth types of urban bare lots are far from densely populated areas of human activities. Large areas of bald hills and bare lots formed by resource exploitation and landfill can be used for urban agriculture [64, 65], ecological restoration [5], and biodiversity protection (such as urban wetlands) [66].

Selecting the urban bare lots in 2017 as the target of the study and using the above classification method, we developed a comprehensive list of indexes, such as location, topography, landscape, traffic convenience, service facilities and urban planning class (stipulation of urban planning for the regional land use type), and used them to evaluate urban bare lots in Mianyang City.

The urban bare lots types were divided into five categories: community use (Ⅰ); urban greening infrastructure (Ⅱ); urban infrastructure and public service facilities (Ⅲ); urban agriculture development (Ⅳ); and ecological restoration (Ⅴ). The indicators in the comprehensive evaluation were analyzed quantitatively using a 5-point Likert scale. Its threshold value and quantification standard are based on ARCGIS10.7 spatial analysis, POI (Point of Information) density analysis and other statistical analyses. All urban bare lot patches are compared with the mean and standard deviation of each indicator, and are categorized into five levels corresponding to the five different transformation and utilization types. Finally, the urban bare lots in the study area in 2017 were evaluated and analyzed based on the above procedure.

## Analysis and results

### Spatial distribution and variation in areas of urban bare lots

We extracted the LULC change information at different points in time by hierarchical classification using Landsat TM/OLI series remote sensing images and analyzed the spatio-temporal distribution characteristics of urban bare lots from 1999 to 2017. Fig 2 shows the LULC information in the study area, which is characterized by the expansion of urban built-up area (urban impervious surface in the figure) along the valley floor, forming a similar Y-shape along the Y axis inclined to the northwest. In contrast, the urban bare lots were concentrated in old urban reconstruction areas, new urban development areas, urban-rural transition zones, and both sides of new transport lines being built, such as highways and high-speed railways, which is consistent with existing research [67]. The results indicate that urban bare lots are closely related to human activity intensity; the greater the intensity of human activity, the more urban bare lots.

**Fig 3:**
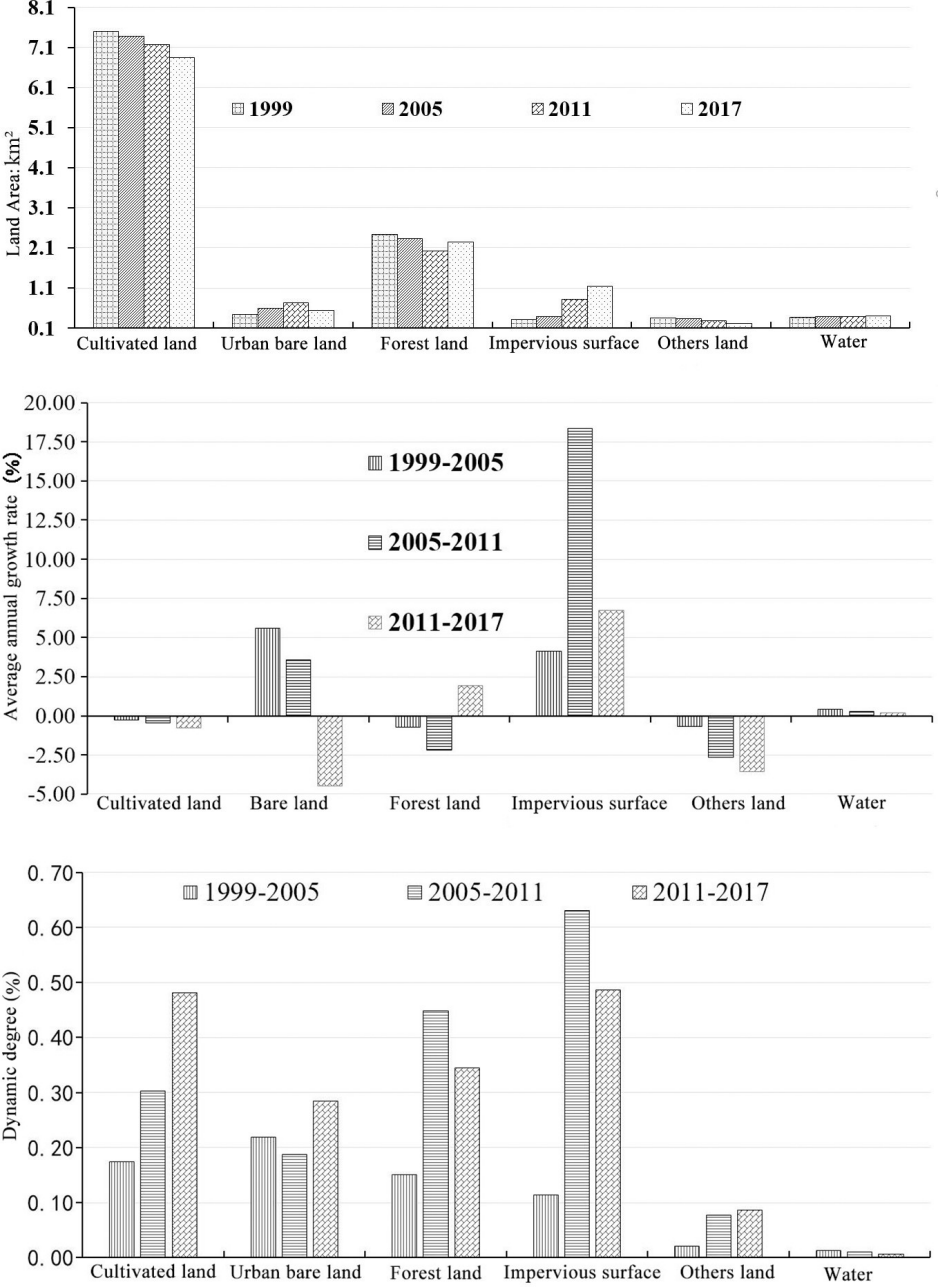
The change of area (a), annual average growth rate ki (b) and growth intensity Di (c) of LULC in the study area from 1999 to 2017.

According to land use type data at different periods, the study area was mainly composed of cultivated land, urban bare lots, forest land, and impervious surfaces, which accounted for approximately 90% of the study area. The cultivated land and forest land accounted for approximately 60% and 20%, respectively. The highest proportion of urban bare lots in the study area was 6.34%, in 2011, and the lowest was 3.91%, in 1999. Water area and other land use types accounted for approximately 6% of the study area on average over the entire period. Overall, urban impervious surface increased notably, while cultivated land decreased. Forest land declined first (1999–2011) and then increased (2011–2017), while surface water basically remained stable (Fig 3(A)).

From 1999 to 2017, the urban impervious surface area exhibited the highest growth rate, with an average annual growth rates of 4.12% in 1999–2005, 18.37%, in 2005–2011, and 6.74% in 2011–2017, while the growths rate of urban bare lots in the same periods were 3.57%, 5.58%, and -4.46%, respectively. The most notable changes were associated with urban impervious surface, followed by cultivated land, forest land, and urban bare lots (Fig 3(B) and 3(C)).

### Urban bare lot distribution and transformation trends

#### Topographic distribution

DEM (Digital Elevation Model) analysis was used to obtain slope, aspect, and elevation data (Table 3). Urban bare lots were mainly distributed over an elevation interval of 40 m (460–500 m). The lowest and highest urban bare lot elevations in 1999 were 372 m and 623 m, respectively, with an average of 466 m. By contrast, the lowest and highest elevations of urban bare lots in 2017 were 411 m and 654 m, respectively, with an average of 495 m. The lowest, highest, and average elevations of urban bare lots all exhibited upward trends. Since urban bare lot distribution reflects human activity intensity, the results demonstrate that human activities are gradually being extended to higher terrain.

**Table 3:** Slope, aspect, and DEM for urban bare lots.

		1999	2005	2011	2017
SLOPE (%)	0.00–3.02	18.75	20.09	22.77	21.10
	3.03–6.37	35.99	37.36	39.92	38.54
	6.38–9.73	23.55	23.24	22.32	23.01
	9.74–13.42	12.86	12.20	10.25	11.28
	13.43–17.78	5.79	5.14	3.61	4.48
	17.80–23.83	2.35	1.63	0.98	1.29
	23.84–49.00	0.71	0.34	0.16	0.29
ASPECT (%)	Flat	0.10	0.11	0.13	0.12
	North	12.25	11.73	12.46	11.65
	Northeast	12.44	12.86	13.02	11.87
	East	11.71	12.14	12.22	11.89
	Southeast	12.77	13.12	12.81	13.03
	South	14.00	14.98	13.51	14.63
	Southwest	13.59	13.45	13.04	13.95
	West	11.71	11.04	11.39	11.68
	Northwest	11.42	10.58	11.42	11.17
DEM(m)	Min	410.00	372.00	411.00	411.00
	Max	640.00	623.00	633.00	654.00
	Mean	497.08	466.77	489.21	495.14

More than 65% of the urban bare lots were distributed on slopes with gradients less than 8%more than 40% of the urban bare lots were distributed on slopes between 3–8%, and only 5% of the urban bare lots were distributed on slopes greater than 15%, which reflected the obvious hilly characteristics in the study area.

#### Urban bare lots land use change characteristics

The landscape index was used to analyze the variation in the number, size, and shape of urban bare lots (Table 4). With regard to changes in patch number (*PN*) and average patch area (*MPA*), *PN* was the lowest (1558) in 2011 and the highest (2430) in 2017, while *MPA* was the highest (46,030 m^2^) in 2011 and the lowest (21,674 m^2^) in 2017, indicating an inverse trend. Secondly, in terms of the bare mean shape index (*MSI*), *MSI* was the highest (1.81) in 2011 and the lowest (1.29) in 2017, while the dispersed degree of patch shape (*MSISD*) was the highest (0.72) in 2005.

**Table 4:** Landscape indices for urban bare lots in the study area from 1999 to 2017.

	1999	2005	2011	2017
*PN*	1697	1592	1558	2430
*MPA / m*^*2*^	26166	37372	46030	21674
*Max*.*PA / km*^*2*^	1.50	8	2.41	1.13
*MPASD / km*^*2*^	0.069	0.219	0.138	0.067
*MSI*	1.67	1.63	1.81	1.29
*MSISD*	0.65	0.72	0.68	0.61

The mode of growth of urban bare lots was mainly via patch expansion and merging before 2011, while urban bare patch fragmentation became the dominant mode of growth after 2011. Consequently, the number of bare patches increase during this period, although both the total area and maximum patch area of urban bare lots exhibited downward trends, which was consistent with the actual status on the ground. Under the joint influence of urban size expansion and peri-urban road construction, a large number of urban bare lots were distributed in the city development zone (such as Yuanyishan District, an economic and technology development area) and on both sides of newly built roads before 2011. The average annual growth rate (17.8%) and corresponding dynamic degree (0.64%) of urban impervious surface during the same period exhibited the most dramatic changes over the entire study period. With the completion of the construction of expressways around the city and within the study area (such as the Beijing-Kunming Expressway), urban space expansion has become the main cause of urban bare lot formation since 2011. However, the scale has sharply decreased, and the average annual growth rate of urban impervious surfaces (6.8%) and its corresponding dynamic degree (0.48%) has also decreased notably when compared to the rates in the preceding period.

### Land transfer characteristics of urban bare lots

The land transfer matrix of the study area in different periods was obtained using GIS, which revealed the conversion relationship between urban bare lots and other land use types in 1999–2017 (Table 5). Over the past 18 years, 61.35% of urban bare lots were derived from cultivated land and 23.58% from forest land. Over the same period, 51.88% of the urban bare lots was converted into cultivated land, 24.7 9% was converted into urban impervious surfaces, 9% was converted into forest land, and less than 7% was converted into water area and other land use types. Notably, in the three periods between 1999 and 2017, there were significant differences in the rates of conversion of urban bare lots to urban impervious surfaces, which were 5.25% in 1999–2005, 29.77% in 2005–2011, and 27.68% in 2011–2017.

**Table 5:** Land transfer matrix in 1999–2017.

	1999						
		Cultivation land	Urban bare lots	Forest land	Urban impervious surface	Others	Water area
2017	Cultivation land	46.3	3.25	17.98	5.51	1.47	0.63
	Urban bare lots	2.31	0.36	0.4	1.08	0.09	0.21
	Forest land	16.75	1.25	3.48	1.73	0.5	0.39
	Urban impervious surface	0.53	0.06	0.05	2.44	0.01	0.01
	Others	2.11	0.2	0.49	0.45	0.08	0.12
	Water area	0.59	0.17	0.15	0.18	0.03	2.53

In conclusion, cultivated land accounted for more than 50% of the transfer into and from urban bare in the different periods from 1999 to 2017, while the conversion of urban bare lots between urban construction land and forest land accounted for less than 40% of the total urban bare lots. In the process of urbanization, the source of urban bare lots is mainly cultivated land and forest land, while the conversion of urban bare lots is mainly into cultivated land followed by urban impervious surfaces.

### Classification and utilization of urban bare lots

Classification criteria are derived from literature reviews regarding urban land use and land renewal issues since the criteria are developed from the review of cases in general [68–70], although further refinement is required to accommodate the constraints and priorities of local contexts.

#### (1) Site location

On both sides of urban road is one of the main distribution areas of urban bare lots in various countries [23]. The transformation and utilization of bare land in this area has attracted more attention from the government, developers, and community residents. Therefore, the closeness of the urban bare lots to urban arterial roads, rivers, parks, and other geographical conditions conducive to renovation, renewal and utilization influences their social service value and their renewal direction.

#### (2) Topographic

Rugged mountains and steep slopes greatly affect urban bare lot renewal potential [24]. Urban bare lots in flat terrain have better redevelopment prospects due to perceived lower financial and time costs, in addition to the associated uncertainties. In addition, the restoration of bare land in steep slopes has implications for soil, water, and ecological conservation.

#### (3) Landscape characteristics

First, urban bare lot sizes in certain cities can influence their exploitation [71]. For example, community gardens, small market gardens, mini-farms, or urban farms are suitable in parcel sizes of >0.25 ac (0.1 ha), 0.25–1 ac (0.1–0.4 ha), 1–5 ac (0.4–2.0 ha) or >5 ac (2.0 ha), respectively, in Oakland [72]. Secondly, landscape spatial connectivity influences the impact of urban bare lots on adjacent land patches during the transfer process. To reduce the degree of landscape fragmentation and improve the efficiency of urban bare land reconstruction, land use types closest to the urban bare land lots should be considered.

#### (4) Transportation convenience

Transportation-related sites is a main category of vacant land [15], in which spaces are related to transportation systems: e.g., railroad tracks, highways, and conservation areas. Moreover, as most city governments in China are competing to create better urban image to attract investment and residents, areas adjacent to city centers and main roads have great potential for redevelopment [23].

#### (5) Service facility

The comprehensiveness of service facilities can reflect community income, which is closely related to the distribution of urban bare lots. Urban bare lot exploitation practices are considerably associated with neighborhood income levels. In particular, unused lots (33%) tend to be located in neighborhoods with relatively high population densities and low median household income levels [18]. In addition, the availability of water, gas, and sewage infrastructure influences on the overall investment required.

#### (6) Policy orientation of urban planning

Existence of financial/tax incentives attracts and encourages potential developers to set up investments at vacant sites. Moreover, government interested in creating a better urban image encourage and facilitate the redevelopment of urban bare lots in highly visible areas adjacent to urban centers and main roads [23].

**Fig 4:**
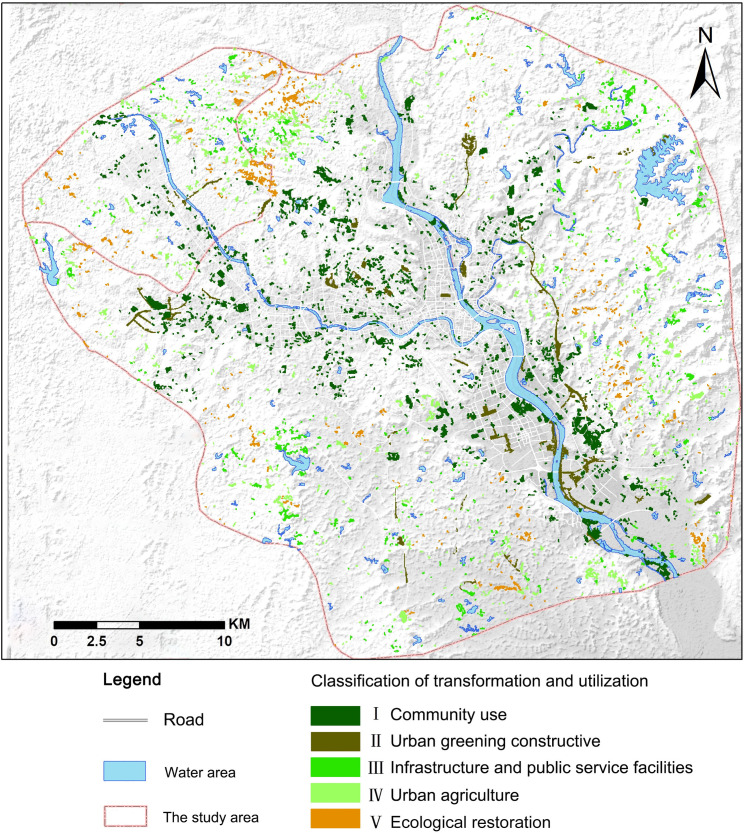
The classification and utilization of urban bare lots in the study area in 2017.

We have established a standard classification using an evaluation index system (Table 6) established based on the five major types of urban bare lot transformation and utilization, to classify and map urban bare lots in 2017, based on zoning (Fig 4).

**Table 6:** Classification and index system for evaluation of transformation and utilization of urban bare lots.

First level index	Second level index	Standard classification and utilization of urban bare lots				
		Community	Urban greening	Urban infrastructure and public services facility	Urban agriculture	Ecological remediation
Location	Distance from the main road of the city (*C*_*1*_)	(0, 100]	(100, 477)	[477, 1968)	[1968, 3462)	≥ 3462
	Distance from rivers and wetlands (*C*_*2*_)	(0, 150)	(150, 547)	[547, 1204]	[1204, 1862]	≥ 1862
	Distance from parks and green spaces (*C*_*3*_)	[0, 10]	[10, 1043]	[1077, 2697]	[2697, 6482]	≥ 6482
Terrain	Elevation (*C*_*4*_)	[0, 425]	[425, 471]	[471, 518]	[518, 564]	≥ 564
	Slope (*C*_*5*_)	[0, 3]	[3, 8]	[8, 15]	[15, 25]	[25, 90]
	*Aspect* (*C*_*6*_)	Flat	South	Southeast-southwest	Northeast-north	Northwest-north
Characteristic of landscape pattern	Patch area (*C*_*7*_)	[0, 900]	[900, 3600]	[3600, 11674]	[11674, 66987]	≥ 66987
	Patch shape (*C*_*8*_)	[0.74, 1.00]	[1.00, 1.29]	[1.29, 1.47]	[1.47, 1.84]	≥ 1.84
	Patch property (*C*_*9*_)	Green	Commercial service land, residential land	Municipal land, roads	Water area and others	Industrial land and storage land
Transportation convenience	Density of public transit stations (*C*_*10*_/km^2^)	> 1285	[1285, 684]	[684, 202]	[202, 10]	≤ 10/ km^2^
Service facility	Density of living and commercial service facilities (*C*_*11*_ /km^2^)	> 2991	[2991, 2187]	[2187, 383]	[383, 5]	≤ 5
	Density of recreational and sports facilities (*C*_*12*_/km^2^)	> 602	[602, 342]	[342, 100]	[100, 0]	0
Urban planning class	Land planning and layout (*C*_*13*_)	Green	Commercial service land, residential land	Municipal land, roads	Water area and others	Industrial land and storage land

The first type (I) of urban bare lots, which is mainly distributed in the built-up area of the city with some lots near the river, covered an area of 22.30 km^2^ with a patch number of 1666 in 2017. This type accounted for 49% and 37% of the total area and numbers of urban bare lot patches, respectively. The second type (II) of urban bare patch covered an area 7.9 km^2^ with a patch number of 110, accounting for 18% and 2% of the total area and number, respectively, of the urban bare lot patches. Because the formation of type II urban bare lots was mainly due to the construction of roads around the city, the type II urban bare lot patches were mostly "strip-shaped". Based on the predominance of the first and second types, they are the main types that experience transformation and utilization in the study area. In addition, the above types are located within the urban built-up area in the valley floor or in the hilly city on the edge of mountains. Such areas are the most intensely affected by human activities and have relatively favorable urban infrastructure. Therefore, such urban bare lots have the greatest potential for transformation and utilization, for example, through the establishment of community green spaces, community gardens, and small parks/playgrounds.

Excluding the type (I) and (II) urban bare lots, the rest of the urban bare lot types were found in areas relatively distant from the urban area. The terrain in the areas was relatively complex. Due to the urbanization process or impact of resource development, degraded areas of hills or "zoned but not built" arable land and abandoned arable land may generate such urban bare lots. Among the three types, the type (Ⅳ) had the largest number and area of the total urban bare lots in 2017, at 26% and 17%, respectively, and were mainly distributed in areas with relatively flat terrain and close to surface water. The type (Ⅴ) was the least, covering an area of only 0.55 km^2^, and accounting for less than 1% of the total urban bare lot area. Type (V) exhibited a scattered distribution and was distant from roads and rivers, posing the greatest challenge in terms of reconstruction. However, it is essential for regional ecological security, because it could be exploited through the ecological restoration of hill sides and exposed surfaces caused by mining and associated municipal waste landfills.

## Discussion

The spatial distribution characteristics of urban bare lots in different periods are "very scattered". The urban bare lots are concentrated in the urban-rural transition zone, urban development zone, and on both sides of the new roads, which is consistent with the findings of other studies [68]. The areas of urban bare lots reached a maximum of 6.34% in 2011 and a minimum of 3.91% in 1999, with an average annual increase of 1.64% in the study area over the study period.

In addition, urban bare lots are distributed mainly in areas with high intensity human activities and newly built-up urban areas, which is consistent with the future spatial expansion of cities with urban sprawl and de-industrialization. Secondly, while they can aggravate urban dust and air particle pollution, as well as cause the severe deterioration of urban ecological environments, if it will not redistribution and re-utilization properly. Therefore, the spatial and temporal distribution characteristics of urban bare lots can be used as key references in urban land adaptability and urban resource carrying capacity evaluation activities.

In terms of topographic features, based on LULC and DEM analysis results, urban bare lots are distributed within the elevation interval of 372–654 m, and 3–8% slopes, which is consistent with the topographic distribution characteristics of impervious surfaces in cities. Studies have shown that [73] a slope not greater than 8%, which is considered a low-slope hilly region, is conducive for development. The spatial distribution and topographic features of urban bare lots highly consistent with the distribution of the built-up areas in the low-slope hilly regions, which further highlights the close relationship between the urban bare lots and human activities–the greater the population size and density, and the intensity of human activities, the more concentrated the urban bare lots distribution [4].

The shift in the landscape patterns of urban bare lots in the study area reflects the spatial growth model of the urban bare lots from a patch scale perspective. The growth in urban bare lot area was mainly dominated by patch boundary expansion and patch amalgamation before 2011, resulting in average patch area increasing gradually during the period. The changes in urban bare patch patterns tended to be dominated by "segmentation" or “fragmentation” after 2011, corresponding to an increasing number of urban bare patches in this period. Both the total area and the maximum patch area of urban bare lots exhibited a decreasing trend.

Therefore, 2011 seems to be a turning point with regard to urban development intensity and land use utilization. Two large new development zones, including Yuanyishan district and Economic and Technological Development Zone, were completed by 2011. In addition, the transit expressway of the Beijing-Kunming Expressway in Mianyang was completely opened to traffic by 2011, in the study area.

The sources of the urban bare lots were primarily cultivated land and forest land, while the urban bare lots were mainly converted into cultivated land, followed by urban impervious surfaces over the study period. The observation indicates high amounts of cultivated land resources around the city may be "zoned but not built" after having been expropriated, and then left vacant to ultimately become urban bare lots, suggesting an inefficient pattern of land use in the study area. More serious is the change in the physical characteristics of the cultivated land derived from urban bare lots, with some of the soil having even been polluted by industry. Therefore, to improve the utilization efficiency of urban bare lots, the scales of the sources of urban bare lots should be first reduced.

Regression models have verified that land-use and the distribution of landscape patterns are significantly correlated with urban resilience [74, 75]. Therefore, we advocate a more proactive approach to urban planning, which would minimize the capture of cultivated and forest lands during urban expansion, in favor of more infill development on urban bare lots, as well as on other urban vacant sites.

There are two types of urban bare lots—community use type and urban greening type—that are both characterized by large areas (jointly accounting for 70% of the total area of urban bare lots) and dense populations. These types have the optimal potential for transformation and utilization. A key step in promoting urban revitalization is the reclamation of functionally obsolete, vacant, and often contaminated sites. Urban bare lots are commonly located in urban areas where basic infrastructure, workplaces and other amenities are already in place and their redevelopment can promote the establishment of walkable neighborhoods, can enhance public transportation, and can revitalize local markets [76]. Additionally, approximately 30% of the urban bare lots are associated with other forms of transformation and utilization. Although the area of urban bare lots classified as ecological restoration type is less than 1% of the total area, they are particularly important for regional ecological security due to their ecological restoration function.

## Conclusions

Vacant lots are widespread persistent phenomena, accounting for a substantial proportion of the urban landscape, and can be strategically exploited in urban development planning and policy initiatives. To inform planning and decision-making processes addressing urban land vacancy, a knowledge base on the characteristics of vacant lots, the types of uses they serve, and their potential use in social–ecological transformation through strategic urban planning and development is required. The quantity, spatial distribution, and land conversion of urban bare lots were analyzed in the present study. The classification method of the urban bare lots prior to their transformation and utilization was explored. A standard classification method was established using an evaluation index system based on the five major types of transformation and utilization of urban bare lots to classify and map the urban bare lots in 2017. The main conclusions are as follows:

1. Urban bare lot spatial and temporal distribution of urban bare lots are closely related to the mode and intensity of human activities in the course of urbanization, which reflects human perception of urban spaces and their ways of transforming natural spaces.
2. Urban bare lots are mainly transformed and exploited as communal and urban green spaces; therefore, urban bare lots have high social, economic, and ecological value.
3. Urban bare lot characteristics in space in the form of landscape patches and other characteristics influence the spatial distribution, classification, and transformation of urban bare lots, which should be taken into account when quantitatively evaluating spatial distribution and formulating reconstruction and utilization schemes.

In summary, before the transformation and utilization of urban bare lots, it is essential to classify to better appreciate their potential beneficial services. Although the evaluation index system proposed in this paper has regional characteristics, it has universal reference value for developing cities in low-slope hilly areas. By assessing vacant lot uses, ecological characteristics and the social characteristics of neighborhoods in which vacant lots are located, planners would be able to more effectively address urban land vacancy, while promoting urban sustainability and resilience.
